# The Buddy-Wire Technique for Impella RP Placement: A Case Report

**DOI:** 10.1016/j.jscai.2025.104112

**Published:** 2025-12-18

**Authors:** Michael V. DiCaro, Vasiliki Tasouli-Drakou, KaChon Lei, Dalia Hawwass, Swetal Patel, Chowdhury Ahsan

**Affiliations:** a Department of Cardiovascular Medicine, University of Nevada, Las Vegas, Nevada; b Department of Internal Medicine, University of Nevada, Las Vegas, Nevada

**Keywords:** cardiogenic shock, case report, interventional cardiology, mechanical circulatory support

## Abstract

Although Impella RP has long been used in acute right ventricular failure, the use of parallel stiff and soft wires (“buddy-wire” technique) for its placement has rarely been described. A 70-year-old man with a left ventricular assist device was found to be in a ventricular tachycardia storm following a firing of his defibrillator. Due to unsuccessful cardioversion, he underwent implantation of an Impella RP. The latter was proven challenging due to the presence of a defibrillator lead; however, the implementation of the buddy-wire technique facilitated its passage, rendering it as a potential solution for patients with complex cardiac anatomies.

## Background

For patients presenting with acute right ventricular (RV) failure, management can include the implementation of microaxial flow devices such as Impella RP as a temporary therapeutic plan for those requiring rapid stabilization and optimization of right-sided filling pressures, especially in the setting of cardiogenic shock. However, patients with complex cardiac anatomy, such as those with tortuous vessels, RV devices, severe tricuspid regurgitation, and annuloplasty rings, may pose a special challenge during the implantation of such devices. One promising solution is the “buddy-wire” technique, which involves the use of parallel stiff and soft wires to enhance device deliverability and stabilize the guiding catheter. To our knowledge, this case is the second reported case in the literature that describes the use of the “buddy-wire” technique in a patient with acute systolic impairment of the RV.^1^

## History of presentation

**Figure 1:**
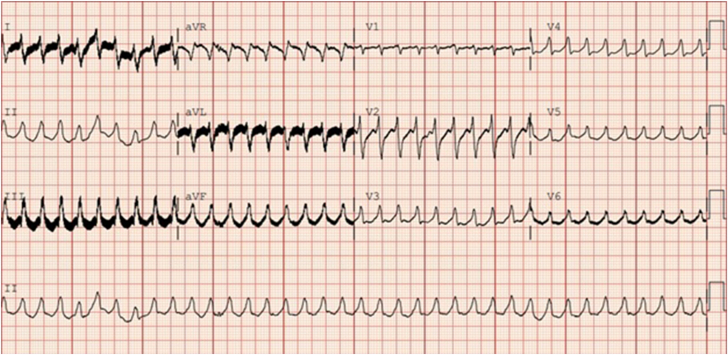
**Electrocardiogramon admission.** Ventricular tachycardia can be seen throughout all leads, with a heart rate of 225 bpm having been recorded.

A 70-year-old man with a medical history of nonischemic cardiomyopathy, heart failure with reduced ejection fraction status-post cardiac resynchronization therapy with defibrillator and HeartMate 3 left ventricular assist device (LVAD) (Abbott), RV failure status-post right ventricular assist device placement with recent explantation, hypertension, diabetes, and chronic kidney disease, presented after multiple shocks from his implantable cardioverter-defibrillator (ICD). On initial presentation, an electrocardiogram (ECG) showed ventricular tachycardia with a heart rate of 200 bpm (Figure 1). Other vitals were within normal limits, with mean arterial pressures in the 90s. He denied symptoms such as dyspnea or chest pain.

**Figure 2:**
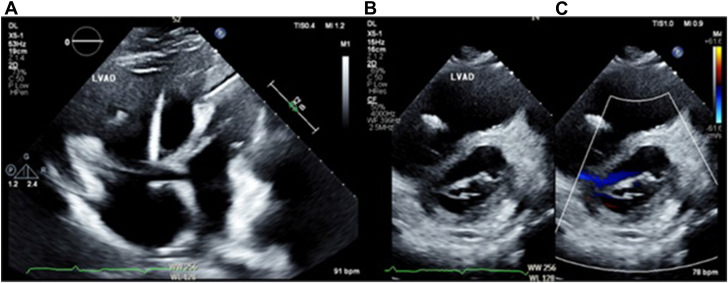
**Pre-Impella implantation transthoracic echocardiogram (TTE) findings.** (**A**) Apical 4-chamber TTE view. Massive RV dilatation concerning for acute RV failure can be seen. (**B** and **C**) Parasternal short-axis TTE view. A D-sign, pressure, and volume overload with an underfilled LV can be discerned.

Due to hemodynamic instability in the presence of ventricular tachycardia, the patient underwent 5 rounds of synchronized cardioversion, received a 150 mg bolus of amiodarone followed by an amiodarone infusion, and was intubated to reduce sympathetic tone, all without success. A bedside echocardiogram was performed, revealing a severely dilated RV with evidence of pressure overload, with an underfilled left ventricle (Figure 2).

Low flow alarms (1.5 L/min at 5100 rpm) from the LVAD were noted and later resolved after the patient was given a 1 L bolus of Lactated Ringer's solution (flow increased to 2.5 L/min). Given the rapidly declining nature of the patient and Society for Cardiovascular Angiography & Interventions (SCAI) stage D cardiogenic shock, he was initiated on vasopressors (dobutamine, norepinephrine, and milrinone). A decision was made by the cardiology team to emergently take the patient to the catheterization lab for right heart catheterization and possible mechanical circulatory support placement.

Right femoral vein access was obtained using a micropuncture kit with ultrasound guidance. An 8Fr venous sheath was placed, and a Swan-Ganz catheter was inserted over the sheath and advanced to the wedge position in the pulmonary artery (PA). The calculated PA pulsatility index was 0.63, cardiac power output was 0.35, and wedge pressure was 23 mm Hg. Due to his impending RV failure, the team proceeded with the placement of an Impella RP for RV-predominant shock in the setting of a pre-existing LVAD. The venous sheath was upsized to a 23F Impella sheath, and the balloon tip catheter was placed into the left PA. The catheter was then removed over a 0.027-inch wire, and the Impella RP was advanced into the inferior vena cava.

**Figure 3:**
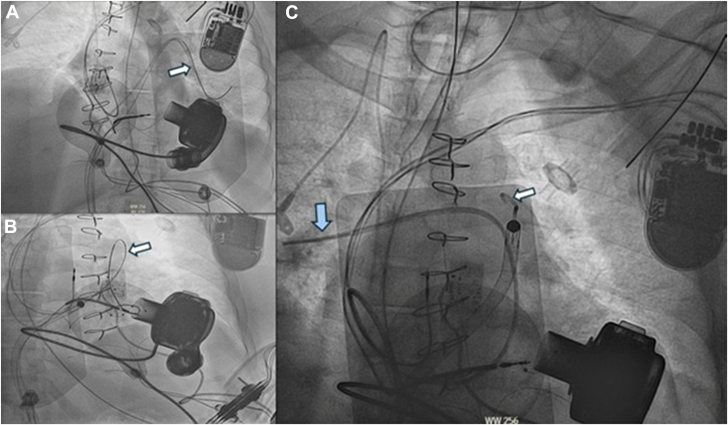
**Right heart catheterization images.** (A) A “Buddy” Swan-Ganz catheter (white arrow) was placed to help facilitate the placement of the back-loaded impella wire. (**B**) The RP Impella was advanced after adequate catheter support, and the initial “Buddy” Swan catheter (white arrow) was removed. (**C**) Final placement of the RP Impella (white arrow). A new Swan-Ganz (blue arrow) was wedged to the right PA for hemodynamic monitoring.

The placement of the Impella RP across the tricuspid valve was proven more challenging than expected, as the guidewire was caught under the ICD lead in a very dilated RV. For this reason, the previously placed guidewire had to be repositioned up, over, and away from the ICD lead (Figure 3A). The buddy catheter technique, akin to the buddy-wire technique, was utilized to facilitate easier passage of Impella RP across the pulmonic valve with the help of the previous Swan-Ganz catheter via the left femoral access. The Impella RP was then successfully placed across the tricuspid and the pulmonic valve, and a new Swan-Ganz catheter was left in place for hemodynamic monitoring (Figure 3B, C). Post-Impella implantation showed improvement of hemodynamics with an increase in pulmonary artery pulsatility index to 1.2, mean arterial pressure to 70 mm Hg, and a decrease in pressor requirements.

The patient was subsequently admitted to the cardiac intensive care unitand transferred to a nearby heart transplant center for further management.

## Discussion

This case describes and illustrates the use of a parallel stiff wire to facilitate the appropriate positioning of an Impella RP in a patient with biventricular failure and complex cardiac anatomy. The use of temporary percutaneous RV support devices, both in the form of percutaneous right ventricular assist device placement and axial-flow pumps,^2^ can be technically challenging in the presence of anatomic obstacles, including pronounced RV dilation, indwelling leads, tricuspid valve interventions, and altered anatomy from previous surgery.^2^, ^3^, ^4^ In these scenarios, advancing the device may be hindered by insufficient guidewire support, wire entrapment, or poor alignment.

The use of a parallel stiff wire, or the “buddy-wire” technique, offers a practical solution to these technical challenges. Currently, this technique is primarily used in challenging percutaneous coronary interventions, where there is a greater need to stabilize the guiding catheter and support the delivery of balloons or stents.^4^ This technique was previously described for Impella RP placement in the context of tricuspid annular ring anatomy.^1^ In the present case, the use of a parallel wire and catheter facilitated smooth passage of the Impella RP by providing additional support, improved alignment, and allowing careful navigation around the indwelling RV lead, thereby preventing lead dislodgement. This approach also reduced the risk of entanglement with the lead, minimized manipulation within the RV, and improved procedural efficiency.

Our experience supports the utility of this method in complex right heart anatomies, particularly in patients with indwelling hardware and severe chamber enlargement. By increasing wire support and stability, the buddy-wire technique may also reduce the risk of complications such as device malposition, tricuspid apparatus injury, or procedural failure. Given the growing population of LVAD and device-dependent patients at risk for acute RV failure, awareness of advanced technical strategies is critical.^5^ Limitations of this approach include the need for operator familiarity with advanced wire techniques and the possibility of wire entrapment.^6^ Nonetheless, in selected patients, the “buddy-wire” strategy may improve procedural success and clinical outcomes.

## Conclusion

In patients with acute RV failure and challenging cardiac anatomy, the buddy-wire technique can facilitate safe and successful placement of percutaneous RV assist devices such as the Impella RP. This case represents only the second published case in the literature and highlights the utility of this technique in scenarios complicated by challenging anatomy. Increased awareness and dissemination of such strategies may improve outcomes for patients requiring temporary RV mechanical support.

## Declaration of competing interest

The authors declare that they have no competing personal or financial interests.

## References

[1] Giannini F., Montorfano M., Jabbour R.J. (2017). Use of a parallel stiff wire to facilitate percutaneous Impella RP ventricular assist device positioning. Cardiovasc Revasc Med.

[2] Pieri M., Pappalardo F. (2018). Impella RP in the treatment of right ventricular failure: what we know and where we go. J Cardiothorac Vasc Anesth.

[3] Attinger-Toller A., Bossard M., Cioffi G.M. (2022). Ventricular unloading using the ImpellaTM device in cardiogenic shock. Front Cardiovasc Med.

[4] Burzotta F., Trani C., Mazzari M.A. (2005). Use of a second buddy wire during percutaneous coronary interventions: a simple solution for some challenging situations. J Invasive Cardiol.

[5] Rajapreyar I., Soliman O., Brailovsky Y. (2023). Late right heart failure after left ventricular assist device implantation: contemporary insights and future perspectives. JACC Heart Fail.

[6] Gutierrez A., Chugh Y., Kostantinis S., Brilakis E.S. (2022). The perils of buddy wire use with a Filterwire. Cardiovasc Revasc Med.

